# Platelet-promoting drug delivery efficiency for inhibition of tumor growth, metastasis, and recurrence

**DOI:** 10.3389/fonc.2022.983874

**Published:** 2022-10-06

**Authors:** Xiaoliang Li, Lanyue Hu, Chengning Tan, Xiaojie Wang, Qian Ran, Li Chen, Zhongjun Li

**Affiliations:** ^1^ Laboratory of Radiation Biology, Laboratory Medicine Center, Department of Blood Transfusion, The Second Affiliated Hospital, Army Medical University, Chongqing, China; ^2^ State Key Laboratory of Trauma, Burn and Combined Injuries, The Second Affiliated Hospital, Army Medical University, Chongqing, China

**Keywords:** platelet membrane, platelet, nanomedicine, delivery system, anticancer therapy

## Abstract

Nanomedicines are considered one of the promising strategies for anticancer therapy; however, the low targeting efficiency of nanomedicines *in vivo* is a great obstacle to their clinical applications. Camouflaging nanomedicines with either platelet membrane (PM) or platelet would significantly prolong the retention time of nanomedicines in the bloodstream, enhance the targeting ability of nanomedicines to tumor cells, and reduce the off-target effect of nanomedicines in major organs during the anticancer treatment. In the current review, the advantages of using PM or platelet as smart carriers for delivering nanomedicines to inhibit tumor growth, metastasis, and recurrence were summarized. The opportunities and challenges of this camouflaging strategy for anticancer treatment were also discussed.

## Introduction

**Figure 1:**
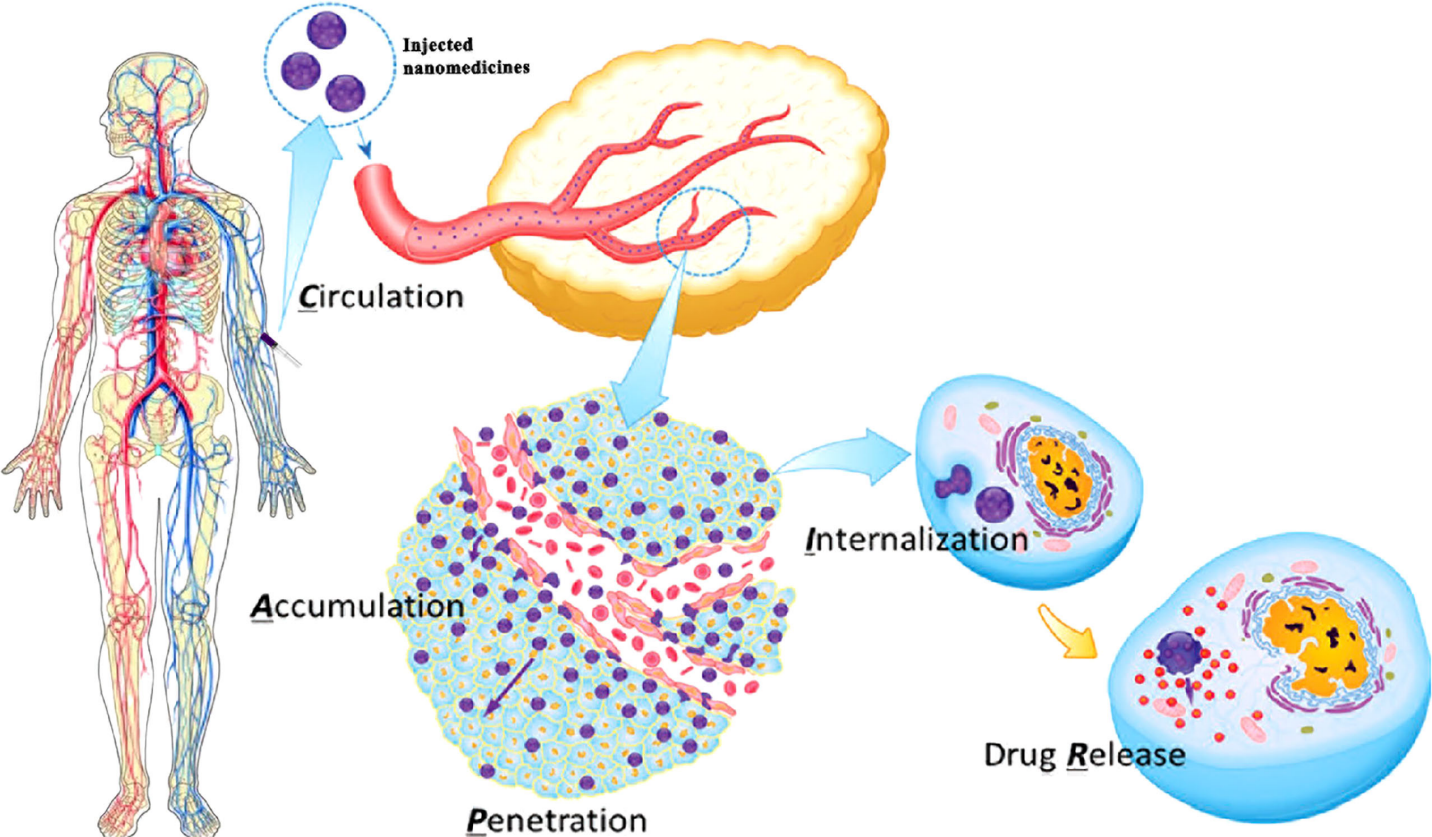
The five-step CAPIR cascade for nanomedicines to deliver free drugs into tumor cells. C step: nanomedicines should be stable and stealthy for long blood circulation, which means nanomedicines should be large (about 100 nm), and the surface should be pegylated and neutral and hide binding groups. A step: nanomedicines should specifically accumulate in tumor site through enhanced permeability and retention (EPR) effect and interaction between the surface ligand on nanomedicines and receptors on tumors. P step: nanomedicines should be small (less than 30 nm) and show a positive charge on the surface for effective tumor penetration. I step: nanomedicines should show positive charge and specific ligands/functional groups on surface to stick to tumor cells for fast cellular internalization. R step: nanomedicines should release the active drug in a “smart” manner modulated by tumor microenvironment (TME) characteristics (5, 6). Figure reproduced with permission (4). Copyright, 2014 WILEY‐VCH (License Number: 5325080186333).

A drug delivery system based on nanoparticles (NPs) is considered a promising strategy for cancer treatment, which could bring an anticancer drug to a specific targeted tumor site, increase the drug concentration in cancer cells, and avert toxicity in normal cells *in vivo* (1). Although nanoparticles could protect the anticancer drug from degradation and clearance in the body, most of the nanoparticles would be removed by the reticuloendothelial system (RES) in the liver and spleen when they are transported through the body after intravenous injection (2). Chan et al. found that only about 0.7% of the intravenous trastuzumab-coated nanoparticles could reach the tumor site in mice (3). The low targeting efficiency caused by nanoparticle–biological interactions is a fatal challenge for the application of nanomedicines in the clinic. For understanding the journey of nanoparticles from the intravenous injection site to the tumor site, the CAPIR (circulation, accumulation, penetration, internalization, and release) cascade rule is illustrated in early research (4), which includes circulation in the blood compartments, accumulation in the tumor, penetration deep into the tumor tissue, internalization by tumor cells, and intracellular drug release (**Figure 1**). Therefore, much higher targeting efficiency for nanomedicines toward cancer cells would be achieved if these nanoparticles efficiently complete the CAPIR cascade (4–6). The nanoparticles that fulfill the rule would be the most excellent drug carriers in the drug delivery system. However, the design of CAPIR-capable nanocarriers is still a tough challenge in the drug delivery field because the required functions for nanocarriers would be different or even opposite in different CAPIR steps (4). For example, the surface of nanomedicines should be neutral, the size should be large, and the stability should be excellent in the circulation step, while the surface of nanomedicines should be positive for effective cell-membrane binding, the size should be small enough for deep tumor penetration, and the nanomedicines could easily be disassembled to release the drug in tumor cell in the other steps (6, 7). Thus, how to design nanomedicines to effectively accomplish the CAPIR cascade is a very hot issue in drug delivery research (6, 8).

Recently, living cells have been considered attractive drug delivery vehicles for cancer therapy due to their high biocompatibility, natural targeting capability, high drug-loading capacity, and good ability to cross biological barriers (9, 10). As shown in the reported papers, nanomedicines used natural cells as carriers could persist in the body for a longer time, specifically target cancer regions and efficiently penetrate solid tumor tissues, thereby perfectly achieving the CAP (circulation, accumulation, and penetration) process in the CAPIR cascade (9–12). Among the living cells, platelet and its membrane showed excellent performance as smart drug carriers for anticancer treatment, especially in the prevention of tumor metastasis and recurrence due to their natural properties (13, 14). Before the PM-coated drug delivery strategy, platelet structure-mimicking drug delivery strategy has also been studied. Materials could mimic the shape and other physical properties of platelets to improve cell recognition in cancer therapy in this strategy (15). However, the synthetic materials could not fully mimic the biofunction of the platelets. Fortunately, PM or platelet camouflaging strategy could solve the bottleneck as scientists expected (16). Herein, literature regarding the platelets in drug delivery systems for inhibition of tumor growth, metastasis, and recurrence was summarized in this review for guiding the nanomedicine delivery system design for excellent clinical translation *in vivo* (**Table 1**). The application of other living cells in drug delivery systems could be seen in other published literature (10, 11, 30).

**Table 1:** PM and platelet for nanomedicine delivery.

Nanomedicine delivery carriers	Therapeutic methods	Therapeutic agents	Release condition	Ref.
PM-coated core–shell nanovehicle	Immunotherapy/chemotherapy	TRAIL/Dox	pH	(17)
PM-coated silica particle	Immunotherapy	TRAIL	─	(18)
PM-coated porous CS-PLGA	Chemotherapy	Bufalin	pH	(19)
PM-coated PLGA	Chemotherapy	DTX	─	(20)
PM-coated PLGA	PTT/chemotherapy	IR780/Dox	pH	(21)
PM-coated liposome	PDT	Ce6	NIR laser	(22)
RGD peptide-modified PM	PTT/chemotherapy	MNPs/Dox	NIR laser	(23)
Platelet	Immunotherapy	aPDL1	Platelet activation	(24)
Liposome-coated platelet *in situ*	Immunotherapy	TRAIL	─	(25)
Platelet	PTT	AuNRs	Platelet activation	(26)
Platelet	Immunotherapy/PTT	R837/NDI-BT	Platelet activation	(27)
Nanodiamond-loaded platelet	Chemotherapy	Dox	Platelet activation/pH	(28)
Platelet	PTT/chemotherapy	IR-820/PDA@Dox	NIR laser	(29)

PM, platelet membrane; CS, chitosan oligosaccharide; PLGA, poly(lactic-co-glycolic acid); NIR, near-infrared; MNPs, melanin nanoparticles; PTT, photothermal tumor therapy; PDT, photodynamic therapy; TRAIL, tumor necrosis factor-related apoptosis-inducing ligand; Dox, doxorubicin.

## Functions of platelet

**Figure 2:**
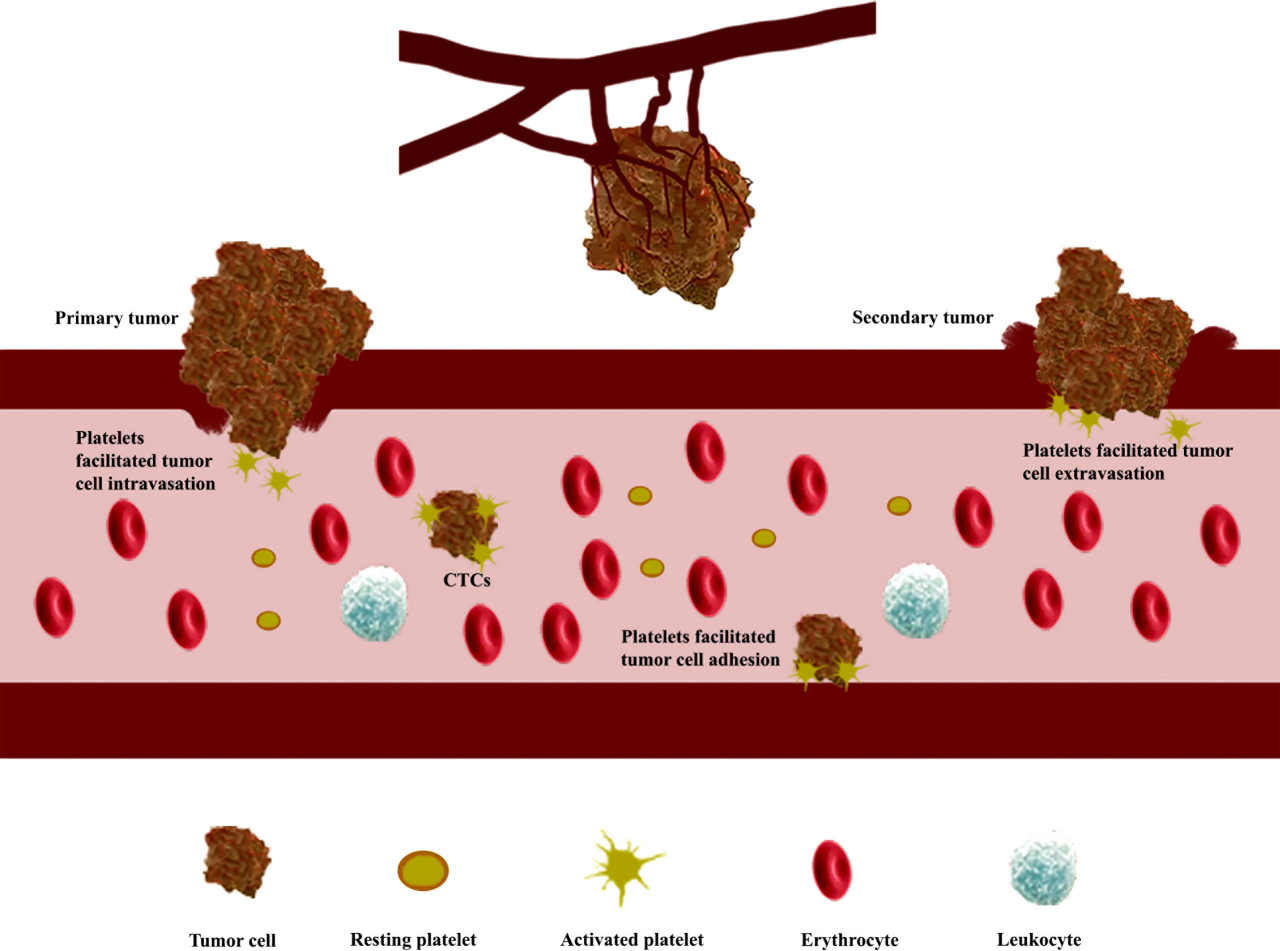
Platelets stimulate tumor angiogenesis, enhance vascular permeability, and promote the formation of metastatic tumors. Platelets stimulate tumor angiogenesis through the release of angiogenesis regulator factors, such as vascular endothelial growth factor (VEGF) and platelet-derived growth factor (PDGF), after activation in the tumor site; platelets trigger epithelial–mesenchymal transition (EMT) activation to enhance vascular permeability of tumor cell; circulating tumor cells (CTCs) binding with platelets are protected from shear stress and immune elimination.

Platelets, derived from megakaryocytes (MKs), are well known for the functions of preventing bleeding, wound healing, and vessel repairing. When the vasculature is damaged or ruptured, platelets could be activated and undergo dramatic shape changes for adhesion to the site of injury, forming a “platelet plug” and blood clotting for hemostasis (31, 32). Regardless of the normal functions, platelets are also known to be involved in tumor angiogenesis (7, 33–35), maintaining tumor vessel integrity (36) and metastasis (37) (**Figure 2**). High platelet counts, related to interleukin-6 secreted by cancer cells stimulating hepatic thrombopoietin (TPO) production (38), are correlated to shorter survival for lung, colon, breast, pancreatic, kidney, and gynecologic cancer patients (39). Although thrombocytopenia could be observed in some cancer patients as well, in the majority of cases, systemic chemotherapy is responsible for thrombocytopenia in cancer patients (40). Furthermore, the function of platelet in tumor growth is still controversial; platelets could inhibit primary colorectal tumor growth but definitely promote metastasis (41). It is well known that metastasis, which accounts for most cancer deaths, is a very complicated and highly inefficient process that detaches tumor cells to different sites through blood vessels and lymphatic vessels (42, 43). During the metastatic progression, platelets would play a very significant role to facilitate the transmigration of tumor cells across endothelium (37) and protect the tumor cells from physical damage and immunosurveillance (43–46). GPIIb/IIIa (also known as integrin αIIbβ3) expressed by platelet could bind to αγβ3 integrin expressed by tumor cells (47) and make αγβ3 active, which could stimulate the NF-κB pathway for epithelial–mesenchymal transition (EMT; an invasive phenotype in tumor cells) activation and induce matrix metalloproteinase (MMP) upregulation for tumor cell invasion (13, 46, 48). After intravasation, tumor cells that entered the bloodstream, known as circulating tumor cells (CTCs), have to overcome several obstacles for survival. Most CTCs would be destroyed or eliminated by shear force and the immune system; only very few CTCs could successfully survive and potentially form metastatic tumors (49–51). Platelets could interact with CTCs through some adhesion molecules such as α6β1, P-selectin, and GPIIb/IIIa; then the adhesive platelets would be activated by several factors including tissue factor (TF), thrombin, or adenosine diphosphate (ADP) secreted from tumor cell (52, 53). Subsequently, the activated platelets could form a thrombus around the CTCs to protect them from shear stress and immunosurveillance in the bloodstream (54). Far more than as shields for tumor cells, platelets could also transfer major histocompatibility complex class I and growth factor β (TGF-β) to tumor cells to prevent natural killer cell recognition and inhibit natural killer cell antitumor reactivity, respectively (45, 55, 56). More detailed information for platelet promotion of tumor metastasis could be found in the reported references (37, 43, 45, 51, 54, 57). Therefore, the interaction between platelets and tumor cells is a very important factor for promoting tumor metastasis, and obviously, it is a good targeting point for metastasis inhibition (58). Furthermore, it is a comparatively simple step from protecting CTCs to protecting circulating nanomedicines in the bloodstream. If the circulating nanomedicines could be protected by platelets, like the way that CTCs were protected by platelets, they could achieve a longer persistence period in the bloodstream (27, 59, 60). In addition, platelets could accumulate around the surgical area due to their inherent function after removing the tumor by surgery (24). Hence, the nanomedicines loaded with platelets could successfully accumulate at the surgical site to prevent tumor recurrence (29). Inspired by these properties, PM and platelets have already been used as excellent drug carriers in delivery systems (59, 60).

## Platelet membrane coating nanomedicines

**Figure 3:**
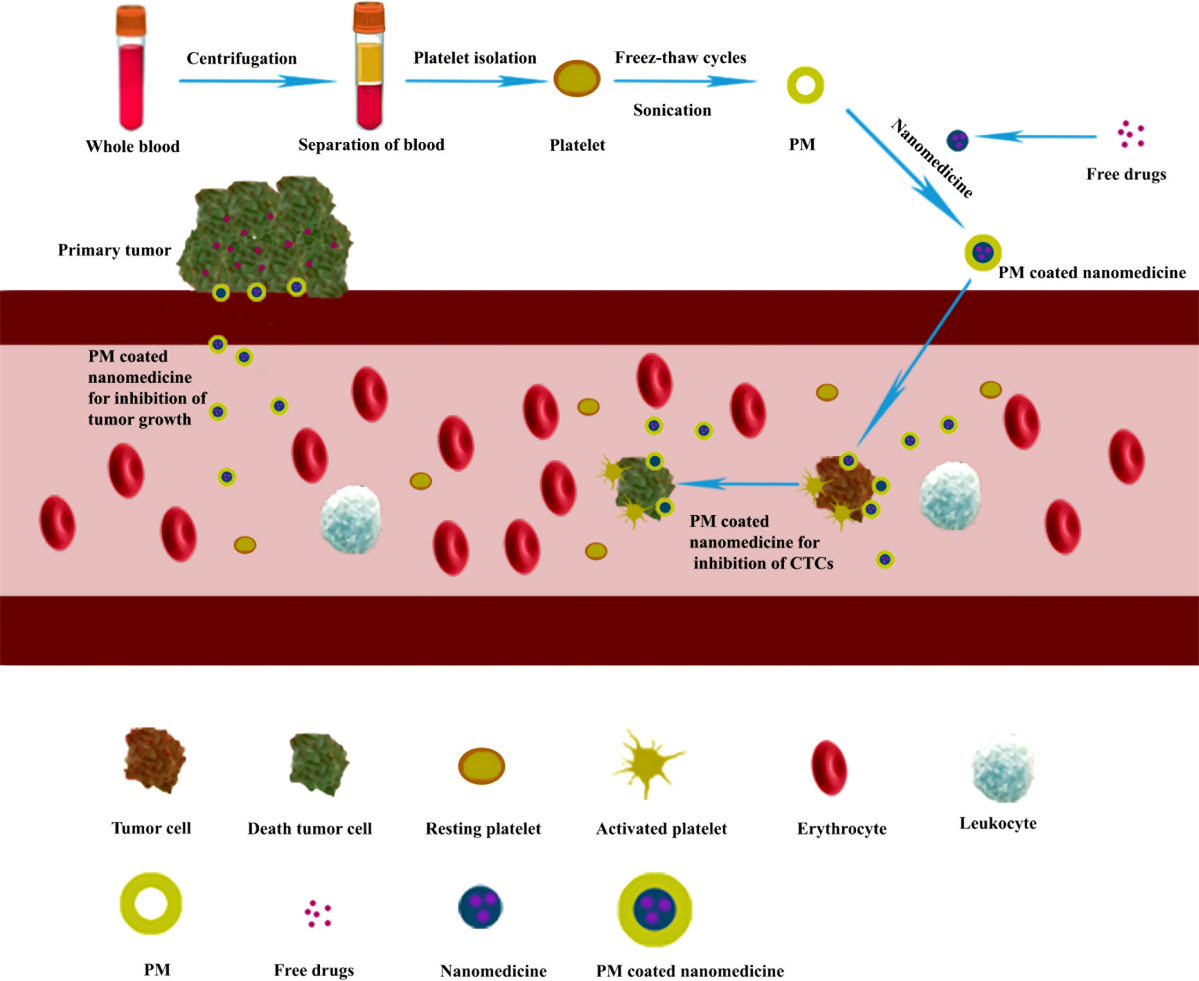
Preparation of platelet membrane (PM)-coated nanomedicines used for inhibiting tumor growth and metastasis. The isolated platelets from whole blood were fragmented by freeze–thaw cycles assisted by sonication and purified by centrifugation to give the desired PM (18). Then the PM fused with nanomedicines to form PM-cloaked nanomedicines for inhibiting tumor growth and metastasis.

Recently, the PM-coating strategy is considered one of the promising techniques for drug delivery in anticancer therapies (14, 61). During the fabrication of PM-cloaked nanoparticles, platelet membrane proteins including immunomodulatory proteins (CD47, CD55, and CD59), integrin components (αIIb, α2, α5, α6, β1, and β3), and other transmembrane proteins (GPIbα, GPIV, GPV, GPVI, GPIX, and CLEC-2) could be translocated or even enriched onto the nanoparticle surface (62). The immunomodulatory proteins could protect nanoparticles from immune surveillance and body clearance by inhibiting macrophage recognition and reducing opsonization in the bloodstream (59); the integrin components could be involved in the adhesion and aggregation during hemostasis; the transmembrane proteins could form the GPIb-V-IX complex to be involved in the initial adhesion of platelets on the subendothelium of damaged blood vessels (63). All these membrane proteins work together to make the PM-cloaked nanomedicines, obtained from nanomedicines encapsulated with PM through different methods such as sonication and extrusion (14), successfully reduce cell uptake and complement activation, target tumor sites, and accumulate around tumor tissues (8) (**Figure 3**). Therefore, the PM-cloaked nanoparticles could be accumulated in tumor tissue by a combination of the enhanced permeability and retention (EPR) effect (64) and high binding affinity between proteins on the PM and receptors on the cancer cells (17). After specifically targeting the tumor site, the lower pH in the tumor microenvironment compared with normal tissues would rupture the PM and release the encapsulated nanomedicines (65).

In 2015, Gu’s group (17) reported the first case that used PM-coated core–shell nanovehicle (PM-NV) bearing two anticancer drugs [tumor necrosis factor-related apoptosis-inducing ligand (TRAIL) for initiation of extrinsic apoptosis signaling (66) and doxorubicin (Dox) for intrinsic apoptosis signaling (67, 68)] for cancer treatment. The PM was obtained from purified platelets (69) maintained in a lysis buffer for 30 min; the Dox encapsulated with NV was prepared by using a single emulsion method with an acid-sensitive crosslinker (70). The synthesized PM and Dox–NV mixture were stirred and maintained overnight, and then the TRAIL was attached to PM through well-known chemical reactions to give the desired PM-coated nanomedicine. After an intravenous injection to mice, the PM-coated nanomedicine showed longer retention time at the tumor site, specific targeting ability on the tumor cells through P-selectin on the PM binding with CD44 receptors on the tumor cell, efficient antitumor efficiency, and CTC elimination. With a similar strategy, TRAIL-decorated platelet membrane-coated silica (Si) particles were reported for the reduction of lung metastasis in a mouse breast cancer metastasis model. Si particles were synthesized with a positive charge on the surface. Purified PM with negative surface charge was incubated with Si particles to form the biocompatible nanoparticles PMDV-Si through electrostatic interaction. Finally, TRAIL was conjugated on the surface of PM based on streptavidin–biotin crosslinking to give the nanomedicines, which could be applied for targeting CTCs in circulation to prevent metastasis in mice (18).

It is known that porous nanoparticles could more efficiently load and release drugs for cancer treatment (71). In 2019, porous poly(lactic-*co*-glycolic acid) (PLGA) polymer nanoparticles coated with PM were reported for anticancer drug bufalin (Bu) delivery (19). The PLGA polymer conjugated to chitosan oligosaccharide (CS) firstly and then mixed with porogen vitamin E polyethylene glycol succinate (TPGS) and Bu to prepare porous bufalin-loaded nanoparticles (CS-pPLGA/Bu NPs) with positive surface charges; finally, CS-pPLGA/Bu NPs were coated with PM to fabricate the PM-CS-pPLGA/Bu NPs for H22 tumor inhibition. The porous NPs could specifically target tumor cells through the binding of P-selectin to the CD44 and effectively release the Bu in the tumor region due to the acid condition. With a similar strategy, PM-coated docetaxel (DTX)-loaded PLGA system had been reported in the same year (20). After intravenous injection into mice, the fabricated nanomedicine showed a long retention time, low toxicity, and good tumor inhibition effect for lung cancer. Moreover, PLGA could simultaneously load chemotherapeutic drugs and photothermal agents for combined chemotherapy and photothermal tumor therapy (PTT) (21). IR780 (72) (photothermal agent) and Dox (chemotherapeutic drug) were loaded into PLGA in one step to construct the IR780@PLGA/Dox and coated with PM (obtained from murine whole blood) to fabricate PM-IR780@PLGA/Dox system. After intravenous injection, the as-synthesized material showed longer circulation time in the bloodstream, low toxicity, good targeting ability, and excellent combined PTT under laser irradiation and chemotherapy for inhibiting the growth of the 4T1 tumors in mice.

PM could also help photosensitizers transport to the tumor vicinity for photodynamic therapy (PDT). Chlorin e6 (Ce6) was loaded into liposome to form Ce6-loaded liposome (Lps/Ce6) (73). PM was mixed with the Lps/Ce6 to fabricate PM/Lps/Ce6 system. After intravenous injection into 4T1 tumor-bearing mice, the PM/Lps/Ce6 showed long retention time in the bloodstream, low toxicity, and specific targeting ability to tumor tissue. After near-infrared (NIR) irradiation, the Ce6 was released in the tumor site, and enough reactive oxygen species (ROS) was generated for efficient PDT treatment for breast cancer (22).

PM could be modified with some biomaterials to improve its targeting property. RGD (Arg-Gly-Asp) peptides could randomly link to the PM proteins to form RGD peptide-modified nanoscale platelet vesicles (RGD-NPVs) that could specifically bind to αvβ3 integrin to improve targeting effect to cancer cells and tumor vasculature (23). For loading anticancer drugs, melanin nanoparticles [MNPs; PTT agent (74)] for binding with Dox through π–π interaction were encapsulated by RGD-NPVs and followed by incubation with Dox to fabricate the RGD-NPVs@MNPs/Dox system. After intravenous injection into mice, the RGD-NPVs@MNPs/Dox could specifically target the tumor vasculature and the resistant tumor cells for chemo-photothermal elimination of resistant tumor cells and tumor vasculature under NIR laser irradiation.

## Platelet loading nanomedicines

**Figure 4:**
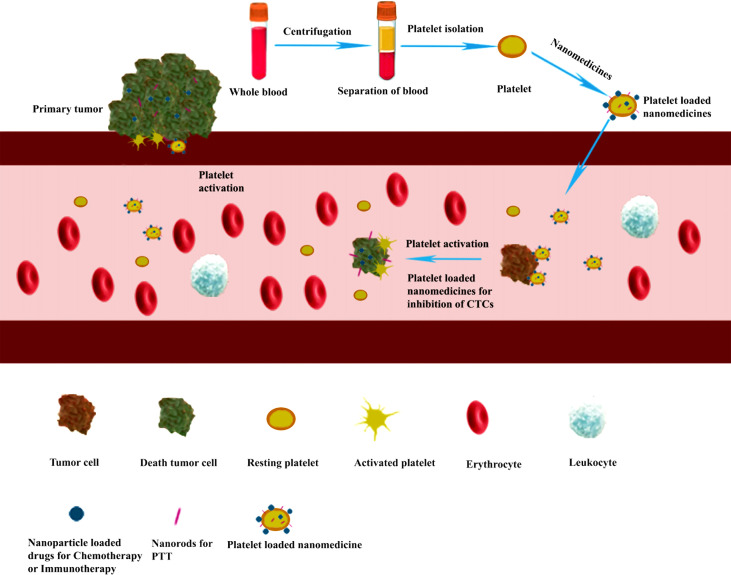
Platelet-loaded nanomedicine for inhibiting tumor growth and metastasis through chemotherapy, immunotherapy, or photothermal tumor therapy (PTT). Purified platelets were obtained from the whole blood by centrifugation. Then the nanomedicines were loaded into the platelet through electroporation methods to form a platelet-loaded nanomedicine system. After intravenous injection, the platelet-loaded nanomedicines would interact with tumor cells through adhesion and activation, and the loaded medicines are released for inhibiting tumor growth and metastasis through chemotherapy, immunotherapy, or PTT.

Platelets could also be used as an excellent drug delivery carrier for targeting and releasing drugs in cancer treatment (**Figure 4**). The medicines could be loaded into the platelet through electroporation (26), incubation (29), or conjugation (75) method. After intravenous injection, the platelet-loaded nanomedicines would specifically be accumulated in the tumor site due to the interactions between platelet and tumor such as tumor cell-induced platelet aggregation (TCIPA), and then intracellular contents would be released after platelet activation (37, 76). Meanwhile, the loaded nanomedicines were released immediately in the tumor site as well. In addition, the vascular damage caused by laser light irradiation in PTT and PDT would also promote the accumulation of the nanomedicines in the tumor site (29).

In 2017, a cancer immunotherapy model using antibodies against programmed-death ligand 1 (aPDL1)-conjugated platelets was reported for the prevention of cancer recurrence and metastasis after surgery (24). The aPDL1 was conjugated to the purified murine platelets through a bifunctional maleimide linker (77) to fabricate the aPDL1-conjugated platelets (P-aPDL1). After intravenous injection of P-aPDL1 into the mice, which had taken the surgery to remove B16 melanomas and triple-negative mammary carcinomas, the P-aPDL1 exhibited longer circulation time in the bloodstream; and aPDL1 would be effectively released at the surgical site through platelet activation and followed by blocking PDL1 on tumor and antigen-presenting cells (APCs) to achieve excellent efficacy for cancer recurrence and metastasis post-surgery. In addition, the same group loaded the P-aPDL1 and chimeric antigen receptor T cells (CAR-T cells) into hyaluronic acid (HA) hydrogel to obtain CAR-T-P-aPDL1@gel system. As the authors expected, the CAR-T cells target the human chondroitin sulfate proteoglycan 4 (CSPG4) and aPDL1 antibodies binding to the tumor cells and blocking PDL1, and the combined CAR-T-P-aPDL1@gel system efficiently prevented the tumor recurrence after surgery in mice (78).

Usually, the platelet-based drug delivery system needs to get purified platelets first and then load them with nanomedicines to fabricate the delivery system. That is not very convenient for clinical applications. King’s group reported that engineered nanoscale liposomes could bind to human platelets *in situ* under physiological shear conditions *ex vivo* (25). The engineered liposomes were obtained from conjugation TRAIL and von Willebrand Factor A_1_ domain (vWFA_1_). The vWFA_1_ could specifically bind to platelet receptor complex GPIb-V-IX (79). Therefore, the engineered nanoscale liposomes would spontaneously bind to platelets and effectively transport TRAIL to target and kill CTCs in flowing blood. The engineered liposomes demonstrated a good potential nanomedicine for preventing cancer recurrence and cancer metastasis in the clinic.

Platelets could not only facilitate the anticancer drugs transport to tumor cells but also efficiently transport photosensitive nanoparticles to tumor tissues for PTT. Gold nanorods (AuNRs) were loaded on murine platelets to prepare the platelet-AuNRs (PLT-AuNRs) with long retention time, specifically cancer targeting and effective photothermal properties. The temperature of the tumor surface could be increased with each laser irradiation after 24 h post-injection of PLT-AuNRs; better PTT effects could be achieved in mouse models with head and neck squamous cell carcinoma (HNSCC). Moreover, the authors found that PLT-AuNRs displayed a better performance than platelet membrane-coated AuNRs in immune evasion and PTT (26). Generally, drug-loaded nanoparticles could exhibit higher loading efficiency and capacity than free chemotherapeutic agents when incubated with platelets (28). Recently, Chen and co-workers reported that Dox was attached to a nanodiamond (ND) surface to construct ND–Dox through the reaction between hydrazone and polyglycerol on ND and then incubated with platelets to fabricate ND–DOX-loaded platelet system (28). After intravenous injection, the ND–Dox-loaded platelets could show longer retention time in the bloodstream, better targeting ability to tumor tissues, lower organ toxicity, and higher chemotherapeutic efficacy than free Dox.

Moreover, platelets could also load different components for the combination of anticancer therapies. In 2021, Ma et al. developed a good anticancer strategy that exhibited combined photothermal immunotherapy for cancer treatment (27). In this study, photothermal nanoparticles (N) were synthesized from naphthalene diimide–bithiophene derivative (NDI-BT) polymer and loaded on platelets (PLTs) with immunostimulator R837 hydrochloride (R) to form the N+R@PLTs system. After intravenous injection into mice, the designed system would show a long retention time in the bloodstream and a high targeting ability to tumor tissues. The tumor could be ablated by PTT under NIR irradiation; then the immunostimulator could inhibit the residual, metastatic, and recurrent tumor tissues. The photothermal nanoparticles and immunostimulator worked together to inhibit tumor growth, metastasis, and tumor recurrence efficiently. More recently, a photothermal therapy agent was loaded together with a chemotherapeutic drug into platelets to achieve combined chemo-photothermal anticancer treatment for inhibition of tumor growth and tumor recurrence (29). In this study, doxorubicin-conjugated carboxymethyl chitosan (CS-g-Dox) polymer (80) was mixed with dopamine to synthesize PDA@Dox nanoparticles through π–π conjugation. IR-820 (81) was loaded into platelets to construct IR-PLT and then incubated with PDA@Dox to fabricate the IRDNP-PLT system. After intravenous injection, the IRDNP-PLT demonstrated long circulation time and good targeting ability and effectively combined photothermal−chemotherapy in 4T1 tumor-bearing mouse models. In addition, the as-synthesized delivery system could significantly inhibit post-surgery tumor recurrence in mouse models.

## Concluding remarks and prospects

Platelet or its membrane camouflaging strategy is a promising approach for targeted nanomedicine delivery in anticancer treatment. Platelets and PM could protect nanomedicines from shear stress and send a “don’t eat me” signal to the immune system for escaping immunosurveillance (82) during the circulation process in the bloodstream. The proteins in the platelet membrane could specifically target tumor cells that effectively enhance the accumulation of nanomedicines in tumor tissues and greatly reduce the off-target side effect of nanomedicines. All these results illustrated that the PM or platelet camouflaged nanomedicines had effectively achieved the CAP process and displayed higher targeting efficiency for anticancer therapy than unprotected nanomedicines. Moreover, combined anticancer therapies could be performed by loading two different agents, for example, loading chemotherapeutic drugs and photothermal agents for combined chemo-photothermal treatment (21). Therefore, the PM or platelet-camouflaged nanomedicines could effectively inhibit tumor growth and prevent tumor metastasis and recurrence *in vivo*.

However, the PM or platelet camouflaging strategy is still far away from the clinical application. For example, 1) the source of the platelet is the first issue that should be considered. Platelets from the donors are not enough to meet patient demand, which greatly limits large-scale applications. 2) The integrity of PM should be considered for PM-coating nanomedicines. The PM structure and protein sequence may be changed, and some fragments would be lost when treated with a lysis buffer during the preparation process, which would result from unexpected side effects in circulation. 3) Platelet activation would cause dramatic shape changes and lead to the release of intracellular content (83). Undesired activation of platelet-related nanomedicines should be avoided in storage and circulation (84). 4) Moreover, the camouflaging nanomedicines that showed long retention time in the bloodstream, high targeting ability to tumor tissue, and deep tumor penetration are still sequestered in the spleen and liver, and only a small portion of nanomedicines would reach the tumor sites (29).

The PM or platelet camouflaging nanomedicines demonstrated long circulation time in the bloodstream, efficient tumor accumulation, and deep tumor penetration for the inhibition of tumor growth, metastasis and recurrence, However, more efforts and multi-field collaborations are required for clinical applications of these camouflaging nanomedicines. It is time to develop easily prepared, visible, and smart carriers to efficiently deliver medicine to tumor tissues. For example, artificial platelets released from human-induced pluripotent stem cell-derived MKs could be a sufficient resource for clinical applications in the near future (85), and we could also expect that, with the help of gene editing and chemical editing, the membrane protein expression level on the artificial platelets would be precisely regulated to let the artificial platelets become smarter for targeting tumor tissues and decorated with fluorescent materials at the same time to make the artificial PM and platelets visible while being monitored during the *ex vivo* generation procedure. As a result, the man-made artificial PM and platelets with bright fluorescence and specific ligands targeting the overexpressed receptors on tumor cells will widely be used as smart carriers for drug delivery. Meanwhile, nanomedicines as well as some tumor microenvironment (TME) modulators will be coated with either artificial PM or artificial platelets to form visible and smart nanosystems for anticancer treatment. After intravenous injection, the nanosystems, which could be visible through fluorescence imaging, would show a long blood circulation to give time for tumor accumulation, because the PM or platelets could protect the nanomedicines from the shear stress and immunosurveillance efficiently. Moreover, the nanosystems would be accumulated in the tumor site due to the EPR and mainly based on the TCIPA and the specific ligand–receptor interaction. As the nanomedicines and TME modulators are released in the tumor site, the TME modulators would normalize the TME to enhance nanomedicine penetration (86), and the nanomedicines would penetrate the tumor easily and become positively charged as acidity increases, efficiently triggering fast cellular uptake. As a result, the combination of state-of-the-art nanotechnology, biotechnology, and material science would achieve efficient chemotherapy, immunotherapy, PTT, PDT, or combined therapies by using PM or platelet camouflaging strategy for anticancer treatment.

## Author contributions

XL: writing–review and editing. LH: writing—review. CT: writing—review. XW: writing—review. QR: supervision. LC: supervision and editing. ZL: supervision and editing. All authors contributed to the article and approved the submitted version.

## Funding

This work was supported by grants from the National Natural Science Foundation of China (82020108025, 81770197, and 81903838), Young talents program of Chongqing (T03010008), and Natural Science Foundation of Chongqing, China (cstc2020jcyj-msxmX0051 and 2022NSCQ-MSX4384).

## Conflict of interest

The authors declare that the research was conducted in the absence of any commercial or financial relationships that could be construed as a potential conflict of interest.

## Publisher’s note

All claims expressed in this article are solely those of the authors and do not necessarily represent those of their affiliated organizations, or those of the publisher, the editors and the reviewers. Any product that may be evaluated in this article, or claim that may be made by its manufacturer, is not guaranteed or endorsed by the publisher.
